# Venous Free Flap with Interposition Bypass Graft for Arteriovenous Fistula Preservation: A Case Report

**DOI:** 10.1055/a-2122-6029

**Published:** 2023-10-12

**Authors:** Cyril Awaida, Marion Aribert, Natalie Weger, Kendall Keck, Andrei Odobescu

**Affiliations:** 1 Division of Plastic and Reconstructive Surgery, University of Montreal Hospital Center, Montreal, Quebec, Canada; 2 Department of Surgery, University of Iowa Hospitals and Clinics, Iowa City, Iowa; 3 Department of Plastic Surgery, University of Texas-Southwestern, Dallas, Texas

**Keywords:** arteriovenous fistula, saphenous vein, squamous cell carcinoma, venous free flap

## Abstract

Cutaneous squamous cell carcinoma (CSCC) overlying an arteriovenous fistula (AVF) is rare and presents unique challenges. This case report describes a method of fistula preservation after CSCC excision using a flow-through venous free flap. The saphenous vein of the venous flap was used as flow-through segment for AVF preservation. The flap was inserted along the dorsal aspect of the forearm wound and microvascular anastomosis of the arterial inflow was completed using a vein just proximal to the radiocephalic fistula anastomosis. Venous outflow was established by creating an end-to-end vascular anastomosis between the cephalic vein and the greater saphenous vein. A separate subcutaneous vein was used to provide a low-pressure outflow for the flap to avoid congestion. This case demonstrates an option for AVF preservation that has not been previously described. It also highlights the importance of a multidisciplinary approach for the safe treatment of CSCCs overlying AVFs.

## Introduction

In recent years there has been significant improvement in patient survival, overall health, and quality of life after solid organ transplant. However, chronic immunosuppression places patients at risk for several complications including malignancy. 1 In fact, cutaneous squamous cell carcinoma (CSCC) remains the most common cutaneous cancer in transplant patients and one of the leading causes of morbidity and mortality. 2 CSCC overlying an arteriovenous fistula (AVF) is a rare occurrence and presents an additional clinical challenge. 3 These patients require expeditious recognition and treatment as invasion of the underlying AVF by the tumor may leave them at risk of life-threatening bleeding and loss of functional access. 4 Treatment goals for these cases include complete surgical resection of the CSCC and AVF preservation. However, patients are often left with sizable soft tissue defects after adequate resection with concern for exposed underlying vital structures. This is especially evident in patients with CSCC of upper extremities where primary closure of the wound is seldom possible. 4 5 6 This poses a reconstructive challenge often requiring microsurgical expertise and free tissue transfer. 6 7 8

Only a few cases of skin malignancy overlying AVF have been reported. 4 We report a case of a renal transplant patient with a CSCC overlying a radiocephalic AVF treated by surgical excision of the lesion with preservation of the fistula using arterialized venous free flap based on the saphenous vein.

## Case

**Fig. 1:**
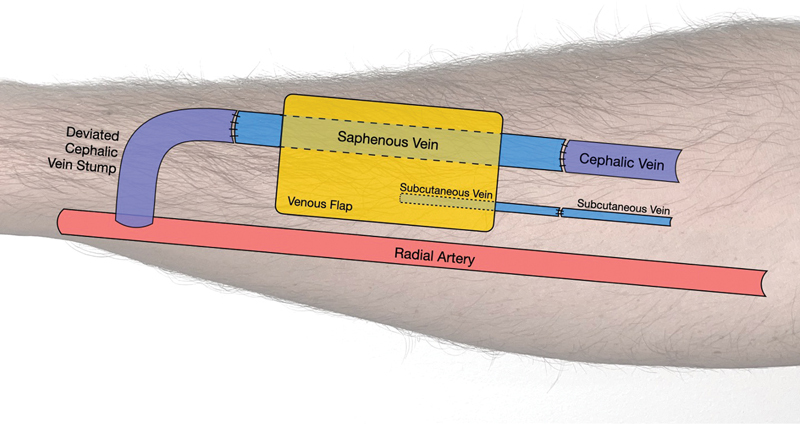
Schematic diagram of flap design. Arterial inflow to the flap is completed using the venous stump of the radiocephalic fistula. The saphenous vein of the venous free flap is used as an interposition bypass graft for arteriovenous fistula preservation and a second subcutaneous vein serves as additional venous outflow. Fistula outflow is established by creating an end-to-end vascular anastomosis between the cephalic vein and the distal end of the greater saphenous vein.

**Fig. 2:**
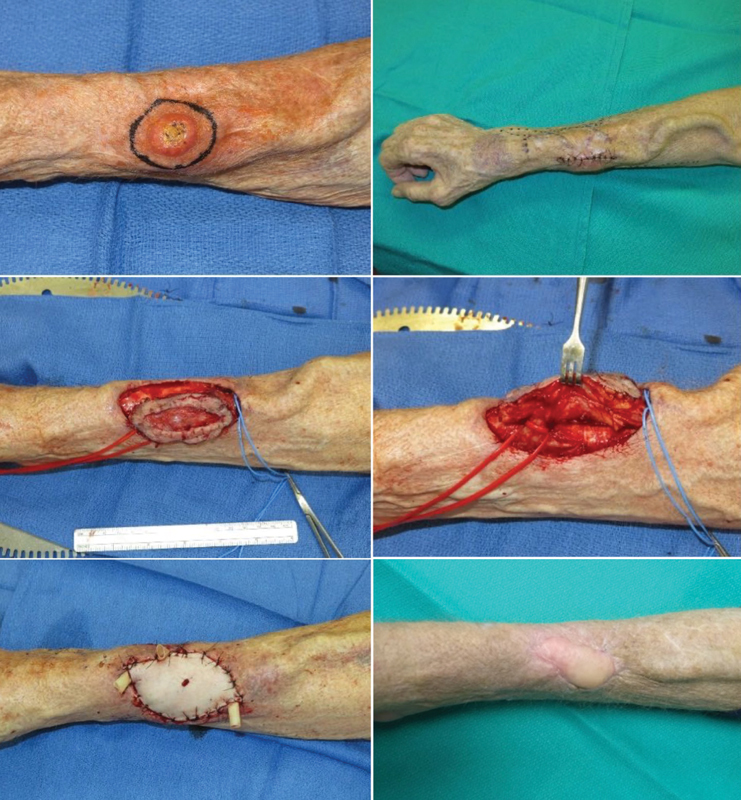
(Above, left) Preoperative photo of the cutaneous squamous cell carcinoma overlying the arteriovenous fistula (AVF) prior to excision. Note the distance to the radial head and location of the tumor in composing much of the distal one-third of the forearm. (Above, right) Preoperative photo of the forearm and AVF after initial excision and prior to further excision of margins and venous free flap. (Center, left) Intraoperative photo during resection of the tumor margins. (Center, right) Isolation of the AVF. The red vessel loop identifies the venous stump of the radiocephalic fistula, whereas the blue vessel loop identifies the cephalic vein. (Below, left) Day of surgery postoperative photo. (Below, right) Postoperative photo during clinic follow-up.

The patient is a 77-year-old man with a history of congenital atrophic kidney disease that required hemodialysis via right distal wrist AVF. Six months after initiating hemodialysis he received a living related renal transplant and had adequate renal graft function to this day. His AVF was functional but not in use at the time of presentation when he was found to have an enlarging cutaneous lesion overlying his AVF measuring 2.5 cm in diameter. Initially, excision of the cutaneous lesion without AVF ligation was performed. The pathology demonstrated well-differentiated invasive CSCC with an invasion depth of 0.4 cm. The deep resection margin was insufficient (less than 0.1 mm). Reexcision with AVF reconstruction using a flow-through venous flap was planned. A 5 cm × 3 cm × 2 cm skin and soft tissue area including the anterior wall of the AVF was resected, and negative margins were confirmed. Given patient's comorbidities, desire to limit donor site morbidity and need for AVF wall reconstruction, a venous free flap based on the saphenous vein was chosen as the preferred reconstructive option. The flap was harvested from the patient's left medial calf to cover the forearm defect. The saphenous vein was used as an interposition bypass graft for AVF preservation. Microvascular anastomosis of the arterial inflow was completed using the venous stump of the radiocephalic fistula for inflow. Fistula outflow was established by creating an end-to-end vascular anastomosis between the cephalic vein and the distal end of the greater saphenous vein. To prevent venous congestion, an additional venous anastomosis was performed ( Fig. 1 ). The venous flap was inset after confirming adequate perfusion. At outpatient follow-up, the patient was noted to have developed necrotic edges on his flap that were debrided and treated with appropriate dressings. The patient's flap healed and the fistula remained functional ( Fig. 2 ). Informed consent was obtained from the patient for this case report.

## Discussion

Very little literature exists on the management of cutaneous malignancies overlying AVFs. A case series written by Lucero et al describes two cases with resection of the overlying cutaneous cancer and linear approximation of the wound without disruption of the fistula. 4 Another report by Nath et al described treatment of small CSCC overlying AVF with radiotherapy only. 9 While this is an option in select cases, radiation therapy efficacy is dependent on the size of the lesion, its histopathologic profile, and its location. It is also known to cause serious complications such as fibrosis and skin necrosis that would potentially lead to AVF exposure and subsequent rupture. 10 Thus, this was not a viable treatment approach in our patient as the CSCC was invasive and required wide local excision per standard of care. 2 However, resection of a cutaneous cancer overlying a superficial AVF presents unique challenges as the risk of potential complications including laceration, hemorrhage, and the need for ligation are high. 4 9 Damage to these fistulas rendering them unusable has consequences in patients who may need them for lifeline dialysis access. While there is ongoing debate in medical community regarding optimal management of AVF in patients after successful renal transplant, most agree with AVF preservation unless there is concern for high output fistula leading to cardiac strain. 11 12 13 14 AVF preservation in our case was important as the patient had a history of multiple hospital readmissions with possible impending renal transplant failure.

**Fig. 3:**
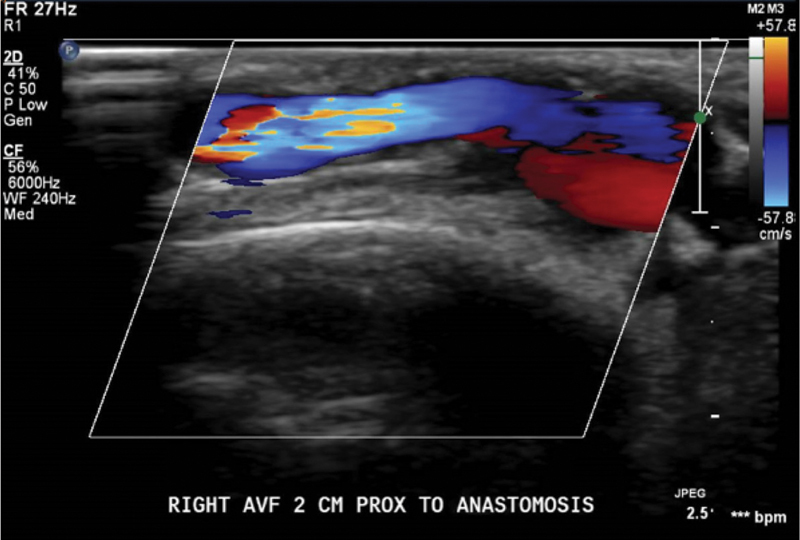
Duplex of the arteriovenous fistula 5 months after reconstruction with the venous flap.

Defect reconstruction using free flaps allow for appropriate coverage with preservation of function but often lead to significant donor site morbidity and require ligation of one of the major arterial branches. 15 Given these constraints venous free flaps were developed to treat burn scars and other wounds in the upper and lower extremities. 16 Venous flaps are readily available, don't require sacrifice of major arterial branch, are pliable, and can be harvested as composite grafts. 17 A new method described in this paper achieved simultaneous goals of adequate cancer resection and large soft tissue defect coverage with AVF preservation using a flow-through free venous flap. This technique showed promising results in our patient who had a functional fistula, healed free flap, and donor sites. His 5-month postoperative duplex ultrasound showed a functional AVF ( Fig. 3 ). The flap showed some marginal loss and delayed healing to the tissues of the forearm. Given the high flow nature of the reconstructed fistula, steal syndrome could have accounted for the poor marginal perfusion within the flap. 6 18 19 20

In conclusion, this case report describes a new method of fistula preservation and soft tissue coverage after resection of an upper limb CSCC using a flow-through venous free flap. It demonstrates an option for AVF preservation that has not been previously described. It also highlights the importance of a multidisciplinary approach for the safe treatment of cutaneous cancers overlying AVFs for best patient outcomes.
